# Sorghum yield prediction using UAV multispectral imaging and stacking ensemble learning in arid regions

**DOI:** 10.3389/fpls.2025.1636015

**Published:** 2025-10-09

**Authors:** Linqiang Deng, Yaoyu Li, Xifeng Liu, Zhimin Zhang, Juanjuan Mu, Shujie Jia, Yuqiao Yan, Wuping Zhang

**Affiliations:** ^1^ College of Software, Shanxi Agricultural University, Jinzhong, China; ^2^ College of Agricultural Engineering, Shanxi Agricultural University, Jinzhong, China

**Keywords:** sorghum yield, UAV multispectral imaging, machine learning, vegetation indices, spatial autocorrelation

## Abstract

**Introduction:**

Frequent droughts and climate fluctuations pose significant challenges to stabilizing and increasing the yields of drought-tolerant crops like sorghum. Accurate, detailed, and spatially explicit yield predictions are essential for precision irrigation, variable fertilization, and food security assessment.

**Methods:**

This study was conducted in the Lifang dryland experimental area in Jinzhong, Shanxi Province, using a sorghum planting experiment. Multispectral imagery and meteorological data were collected simultaneously using a DJI Mavic 3M UAV during key growth stages (seedling emergence, jointing, flowering, and maturity). A “spectral-meteorological-spatial” three-dimensional prediction framework was developed using eight machine learning algorithms. SHAP values and Partial Dependency Plots were used to assess variable importance.

**Results:**

Ensemble learning algorithms performed best, with the Gradient Boosting model achieving an R^2^ of 0.9491 and Random Forest reaching 0.9070. SHAP analysis revealed that DVI and NDGI were the most important predictors. The jointing stage contributed most to prediction accuracy (R^2^ = 0.9454), followed by maturity (R² = 0.9215) and flowering (R^2^ = 0.9075). Yield spatial distribution ranged from 4,291 to 4,965 kg haR^-1^, with a global Moran’s I index of 0.5552 indicating moderate positive spatial autocorrelation.

**Discussion:**

Integrating UAV multispectral data with machine learning methods enables efficient sorghum yield prediction, with the jointing stage identified as the optimal monitoring period. This study provides crucial technical support for precision planting and efficient sorghum management in arid regions.

## 1 Introduction

Accurate crop yield prediction is fundamental to global food security, particularly in arid and semi-arid regions where climate variability and water scarcity pose significant challenges to agricultural sustainability (Hussain et al., 2019). As climate change intensifies and water resources become increasingly scarce, the risks and uncertainties faced by agricultural production in arid regions have significantly increased. Sorghum (Sorghum bicolor), as a drought-tolerant C4 crop with high water-use efficiency, represents a critical food security solution for these vulnerable regions (Comlekcioglu and Simsek, 2011; Liu et al., 2020). However, traditional yield estimation methods, predominantly relying on manual field surveys and empirical models, are costly, inefficient, and inadequate for capturing the fine-scale spatial variability necessary for precision agriculture applications (O’Kelly and Sivakumar, 2014). This limitation is particularly acute in arid regions where yield variability is influenced by complex interactions between water stress, soil heterogeneity, and microclimatic variations, making accurate yield prediction essential for optimizing resource allocation and ensuring agricultural sustainability.

Unmanned Aerial Vehicle (UAV) technology has revolutionized precision agriculture by providing high-resolution, timely, and cost-effective crop monitoring capabilities (Feng et al., 2021; Zhu et al., 2021). Compared to traditional satellite remote sensing, UAVs offer superior spatial resolution and temporal flexibility, enabling detection of fine-scale crop variations that are crucial for precision management decisions. Recent studies have demonstrated the effectiveness of UAV multispectral data in various crop yield prediction applications. Cheng et al. (Cheng et al., 2022) focused specifically on soil moisture estimation under high canopy coverage using UAV multimodal data and machine learning, addressing one of the key challenges in arid region applications where soil-plant-atmosphere interactions are complex. The integration of machine learning algorithms with remote sensing data has emerged as a powerful approach for crop yield prediction, offering advantages in handling high-dimensional, multicollinear data typical of agricultural applications (Chen et al., 2020; Qu et al., 2024).

Machine learning’s strength in agricultural applications lies in its ability to capture non-linear relationships between predictor variables and yield outcomes. Studies have shown that machine learning models built on UAV multispectral data have achieved promising results in crop yield estimation, providing strong decision support for agricultural management (Wigmore et al., 2019; Li et al., 2024). Machine learning’s advantage in remote sensing data processing lies in its ability to adapt to high-dimensional and multicollinear data (Allen et al., 1998). Van Klompenburg et al.’s (Van Klompenburg et al., 2020) systematic review highlighted that ensemble learning algorithms (e.g., Random Forest, Gradient Boosted Tree) perform well in crop yield prediction, demonstrating strong generalization and stability. However, the performance of different algorithms is influenced by factors such as crop type, feature selection, and data quality, requiring customized optimization for specific applications (Ke et al., 2015).

The application of Explainable Artificial Intelligence (XAI) in precision agriculture has gained significant attention as researchers and practitioners seek to understand the decision-making processes of complex machine learning models. In recent years, explanatory machine learning methods, such as SHAP (Shapley Additive Explanations) and Permutation Importance, have emerged to quantify variable importance and their marginal response mechanisms in the mode. PDP (Partial Dependence Plot) and ICE (Individual Conditional Expectation) curves offer visualization support for revealing complex feature interactions, aiding in refining agronomic management thresholds and strategies (Naga et al., 2024). XAI-driven crop recommender systems for precision agriculture represent a significant advancement in making AI models interpretable for agricultural decision-making, providing not only recommendations but also explanations of the reasoning behind their suggestions.

From the perspective of remote sensing features, the vegetation index, a key variable in spectral remote sensing, reflects the physiological and ecological characteristics of crop canopy structure, leaf area index, and photosynthetic efficiency (Ge et al., 2021). Its response sensitivity varies across different crops and fertility stages, making it a crucial input for yield prediction models (Zhu et al., 2020; Cheng et al., 2022). However, the correlation between vegetation indices and yield is influenced by factors such as environmental conditions, growth stage, and background noise. In arid regions, changes in soil albedo and water stress often reduce prediction accuracy (Dass and Bhattacharyya, 2017; Rajanna et al., 2022; Jia et al., 2024). In addition, crop fertility is a key period influencing yield formation, and the contribution of remote sensing features to yield varies significantly across fertility stages. Studies have shown that multispectral features during the pulling and tasseling stages of maize are highly correlated with final yield (Acharya et al., 2019; Maimaitijiang et al., 2020). The use of multi-temporal remote sensing data provides a more comprehensive description of the crop growth process, improving prediction accuracy (Zhou et al., 2017). However, systematic studies on the relationship between remote sensing characteristics and yield at different fertility stages of sorghum are limited, particularly under arid conditions where the formation mechanism is more complex.

Spatial statistical methods, such as Moran’s I and Fast Fourier Transform (FFT), have been widely used to reveal spatial autocorrelation features and the periodic structure of crop yield (Reynolds, 1970; Zhang et al., 2023). These methods can identify high-yielding and low-yielding regions and provide a scientific basis for spatially differentiated agronomic measures, such as variable fertilization and precision irrigation (Eltarabily et al., 2024). However, most current studies focus on static descriptions of yield spatial patterns and lack deep integration of spatial statistical methods with yield prediction models, especially for dry crops like sorghum, where integrated applications need further development.

Despite significant advances in remote sensing and machine learning technologies, several critical technical gaps persist in crop yield prediction, particularly for drought-tolerant crops like sorghum in arid environments. Current UAV-based yield prediction approaches typically rely on single-time point data or simple temporal averaging, failing to systematically quantify the differential contribution of spectral features across critical growth stages. While studies have demonstrated the value of multi-temporal data for crop monitoring, the optimal timing for yield prediction and the relative importance of different phenological stages remain poorly understood, particularly for sorghum under water-limited conditions. Furthermore, existing approaches predominantly focus on either spectral data or meteorological data in isolation, with limited systematic integration of UAV multispectral imagery with concurrent meteorological observations. This fragmented approach fails to capture the synergistic effects of spectral-meteorological interactions that are crucial for accurate yield prediction in variable environments.

Additionally, while machine learning models have achieved promising accuracy in point-based yield prediction, there is insufficient integration of spatial autocorrelation and pattern analysis into the prediction framework. Current approaches treat spatial units as independent observations, ignoring the inherent spatial dependence that could enhance both prediction accuracy and spatial interpolation capabilities. Many existing studies employ “black-box” machine learning approaches without systematic analysis of feature importance and model interpretability, hampering the identification of optimal spectral indices and the understanding of biophysical mechanisms underlying yield formation. Most UAV-based yield prediction studies have been conducted in temperate or irrigated conditions, with limited validation under the challenging conditions of arid dryland agriculture where spectral signatures may be confounded by soil background effects and water stress.

Research specifically focused on sorghum yield prediction using remote sensing and machine learning approaches is relatively limited compared to major crops like corn, wheat, and rice. The drought tolerance and unique physiological characteristics of sorghum present both opportunities and challenges for remote sensing applications. Studies in arid and semi-arid regions have shown that sorghum exhibits distinct spectral characteristics during water stress conditions, requiring specialized approaches for accurate yield prediction. The C4 photosynthetic pathway of sorghum results in different spectral responses compared to C3 crops, necessitating the development of sorghum-specific vegetation indices and prediction models. However, to date, no study has combined multi-stage UAV-based vegetation indices with daily meteorological variables for sorghum yield prediction under the semi-arid conditions of the Loess Plateau.

To address these identified technical gaps, this study develops a comprehensive “spectral-meteorological-spatial” three-dimensional framework for sorghum yield prediction in arid conditions. Our research makes specific contributions to advance the field of precision agriculture in drought-prone regions through systematic quantification of growth stage-specific contributions to yield prediction, identifying optimal monitoring windows for efficient resource allocation. The study presents novel integration of high-resolution UAV multispectral data with concurrent meteorological observations using ensemble machine learning algorithms, specifically optimized for arid conditions and validated across multiple algorithms. We develop a prediction model that incorporates spatial autocorrelation analysis using Moran’s I and frequency domain characterization through FFT to enhance both point prediction accuracy and spatial pattern mapping capabilities. The research implements comprehensive application of SHAP (Shapley Additive Explanations) and Partial Dependence Plot (PDP) analysis to quantify feature importance and reveal biophysical mechanisms underlying spectral-yield relationships in drought-stressed environments.

The practical contributions include provision of actionable insights for variable-rate management strategies in arid sorghum production systems, including optimal timing for remote sensing data collection. The study enables identification of critical growth stages for monitoring, allowing reduction of data collection costs while maintaining high prediction accuracy. Additionally, we generate high-resolution yield prediction maps with comprehensive spatial pattern analysis to support site-specific management decisions and precision irrigation planning.

This research addresses the critical need for accurate, spatially-explicit yield prediction in arid agricultural systems, where traditional monitoring approaches are inadequate for supporting precision agriculture initiatives. The study was conducted at the Lifang dryland experimental area in Jinzhong, Shanxi Province, representing typical loess plateau arid agricultural conditions. The developed framework has broader implications extending beyond the immediate study context, including improved capacity for yield forecasting in climate-vulnerable regions, supporting early warning systems and market stability planning. The research enhances understanding of crop responses to environmental variability in drought-prone areas, informing adaptation planning under changing climate conditions. It provides a scalable decision support framework for variable-rate management practices, including precision irrigation and targeted fertilization in water-limited environments. The methodology is applicable to other drought-tolerant crops such as millet, pearl millet, and cowpea, and to arid regions globally, supporting sustainable intensification in marginal agricultural lands.

This study utilized the Li Fang Dry Farming Experimental Base in Yuci District, Jinzhong City, Shanxi Province, as the study area. Based on UAV remote sensing data, ground sample survey data, and daily meteorological data of sorghum from May to September 2024, a framework for sorghum yield prediction integrating spectral characteristics, meteorological factors, and spatial features was proposed. The research objectives are as follows:

1. Develop a sorghum yield prediction model integrating UAV multispectral data and meteorological data, using multiple machine learning algorithms for comparative analysis to identify the optimal modeling method.
2. Identify key spectral and meteorological variables affecting sorghum yield using explanatory modeling tools such as SHAP values and PDP, and analyze their marginal contributions to the model output.
3. Systematically evaluate the role of remote sensing features at key fertility stages, such as seedling emergence, nodulation, flowering, and maturity, in yield prediction, and determine the optimal timing for efficient monitoring.
4. Based on the prediction results of the optimal model, the spatial distribution of yield was mapped, and spatial clustering and cyclic changes were analyzed using the Moran index and FFT methods to explore the formation mechanism.

This study will provide theoretical support and technical pathways for precision planting, variable management, and intelligent agriculture of sorghum in arid regions. It will also offer a scalable system for multi-source data fusion and spatial modeling methods in crop yield prediction research.

## 2 Materials and methods

### 2.1 Overview of the study area

**Figure 1:**
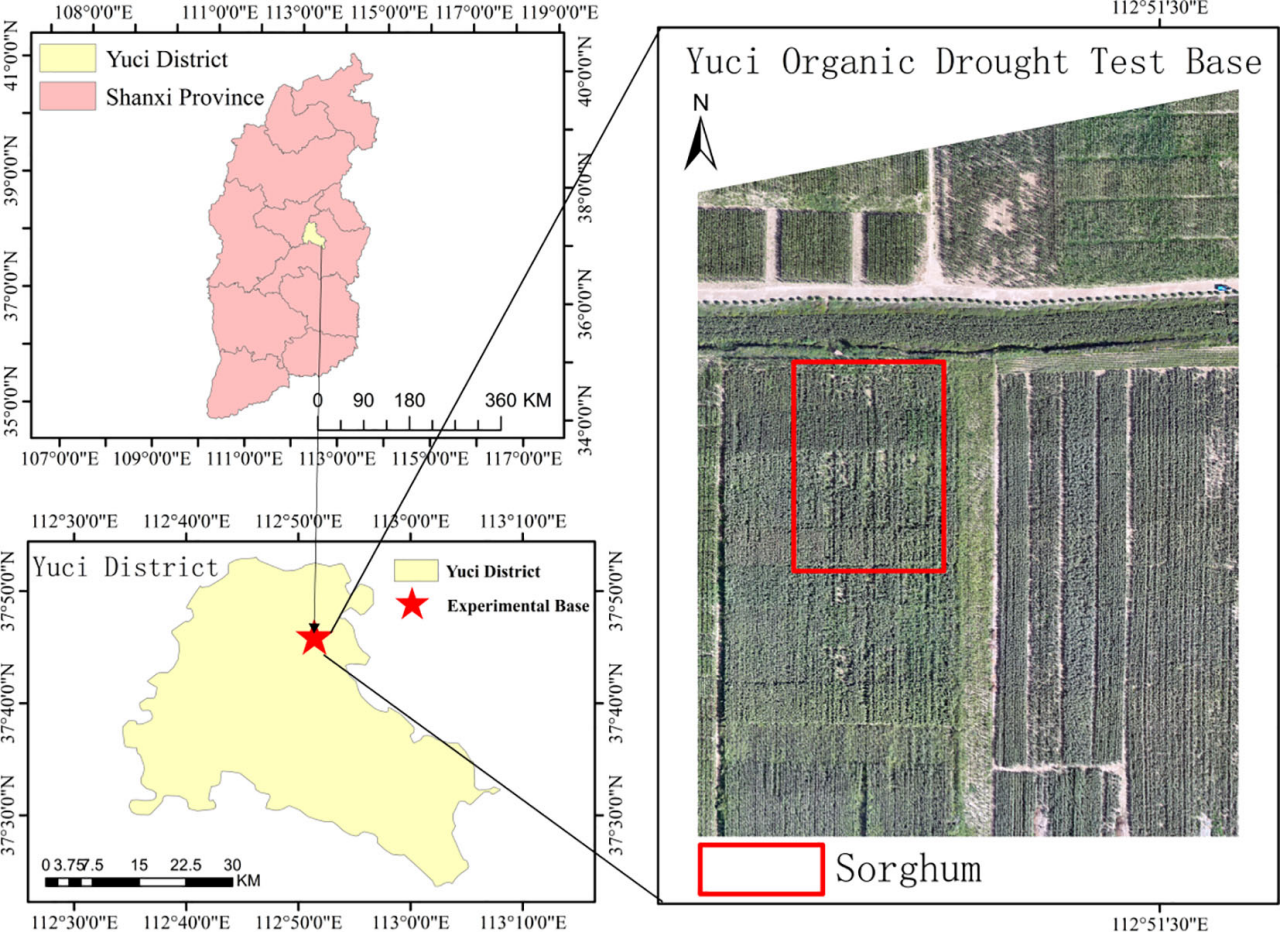
Location of the study area. The maps show the study site at Yuci Organic Drought Test Base located in Yuci District, Shanxi Province, China, with progressive zoom from provincial level (top left) to district level (bottom left) to field level (right). The red box in the satellite image highlights the sorghum experimental plots within the organic dryland farming test base, representing typical Loess Plateau arid agricultural conditions suitable for drought-tolerant crop research.

This study was conducted at the organic dry farming experimental site in Lifang, Yuci District, Jinzhong City, Shanxi Province (N37°51′, E112°45′) (**Figures 1**). The region is situated in the central part of the Loess Plateau, characterized by a typical temperate continental monsoon climate, significant topographic relief, and an elevation range of 767 to 1777 m. Meteorological data over several years show that the regional average annual temperature is approximately 9.8 °C, with a frost-free period of 120 to 220 days. The annual precipitation is around 450 mm, mostly concentrated from June to September. The average annual sunshine hours range from 2000 to 3000, and the annual precipitation is roughly 1.5 times greater than the average annual sunshine hours. The annual precipitation is approximately 450 mm, mostly concentrated from June to September. The average annual sunshine is between 2000 and 3000 hours, and annual evaporation ranges from 1500 to 2300 mm, indicating a characteristic drought-prone climate. The soil in the study area is a mixture of loess and loamy loam, with favorable physical and chemical properties in the tillage layer (0–30 cm). It has an organic matter content of 17.6 g·kg^-1^, a total nitrogen content of 0.98 g·kg^-1^, and a field water holding capacity of 21% (volumetric water content), providing good water retention and fertilizer supply. In May 2024, the sorghum variety “Jinnuo 11” was sown in the study area using mechanized sowing. Plant spacing was set at 15 cm, and row spacing at 45 cm. Basal fertilizer was applied in a single application with N-P_2_O_5_-K_2_O = 12-10–8 kg·ha^-1^ to meet the nutrient requirements for the early stages of sorghum growth.

### 2.2 Data sample collection

**Figure 2:**
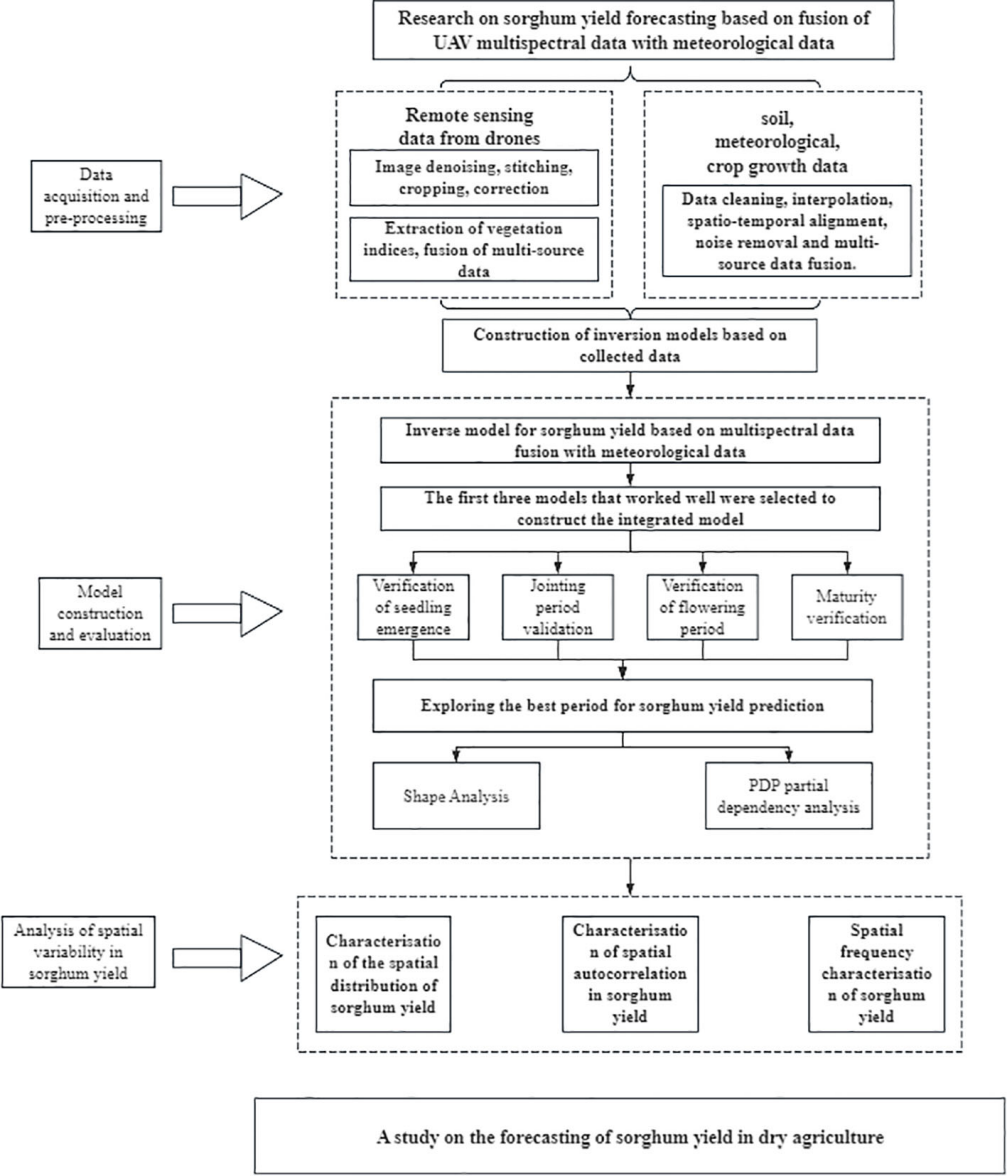
Technology roadmap. The framework consists of three main components: data acquisition and preprocessing (remote sensing data from drones and meteorological data collection), model construction and evaluation (multispectral data fusion with meteorological data, growth stage validation, and optimal period identification using SHAP and PDP analysis), and spatial variability analysis (spatial distribution characterization, autocorrelation analysis, and frequency characteristics). This integrated approach enables comprehensive sorghum yield prediction and spatial pattern analysis for precision agriculture applications.

This study aims to investigate the effects of different fertility stages on yield prediction accuracy during sorghum growth and to develop a sorghum yield estimation model based on multi-source data fusion. The study focuses on analyzing the roles and differences of spectral indices, vegetation indices, and meteorological factors obtained through remote sensing in yield prediction at each key fertility stage. The experiment used a combination of UAV multispectral remote sensing and ground sampling to obtain crop growth information, along with synchronized daily meteorological data collection to comprehensively assess the correlation and modeling capabilities between multi-source variables and final yield. The overall technical approach of the study is shown in **Figure 2**.

### 2.3 Data acquisition and preprocessing

#### 2.3.1 Multispectral data acquisition and processing

During the 2024 sorghum fertility period (May to September), the study employed a DJI Mavic 3M UAV to systematically collect multispectral data across four key phenological stages: seedling emergence, nodulation, flowering, and maturity. The flight altitude was set to 65 meters, and clear weather conditions (10:00-12:00) were selected for execution, with heading and sidetracking overlap rates of 70% and 80%, respectively, to ensure precise geographic alignment. The UAV’s four-channel spectral imaging system captures data in the red (650 ± 16 nm), green (560 ± 16 nm), red-edge (730 ± 16 nm), and near-infrared (860 ± 26 nm) bands. Data are spliced and orthorectified using DJI Terra software, with radiometric correction based on the standard 0.5×1 m gray plate. Spectral reflectance data for each band were then extracted using ArcMap to create a database for calculating the vegetation index.

#### 2.3.2 Measurement of sorghum yield data

The field yield measurement for the study was conducted on September 25, 2024, to obtain accurate sorghum yield data. Eighteen representative plot samples, each with an area of 1 square meter, were selected in the experimental area. The harvested sorghum was air-dried to a stable moisture content, weighed to calculate the yield per unit area, and the resulting yield data were used as target variables for regression modeling and accuracy evaluation with remote sensing and meteorological features.

#### 2.3.3 Meteorological data collection

Daily meteorological data for the area during the sorghum growth period were collected from a small weather station within the experimental station, including parameters such as temperature, humidity, precipitation, and wind speed. The raw data were cleaned and formatted to ensure consistency and reliability. The accuracy of the acquired data was verified by comparing it with data from other meteorological observatories and confirming it with the local meteorological department.

### 2.4 Calculation of the vegetation index

Vegetation indices can indirectly reflect key physiological processes, such as crop photosynthetic efficiency, nitrogen accumulation, and biomass formation, by quantifying canopy spectral characteristics. These parameters exhibit significant synergistic effects with yield components. Considering the varying sensitivity of vegetation indices to specific agronomic traits, this study utilized the UAV’s multispectral sensor channel to establish 16 key vegetation indices (NDVI, RDVI, NLI, GNDVI, RVI, SAVI, NDGI, DVI, OSAVI, GI, MSR, GRVI, CLgreen, WDRVI, TVI, NDWI) (Ke et al., 2015; Naga et al., 2024; Mitra et al., 2025), to identify the optimal yield monitoring metrics, as detailed in **Table 1**.

**Table 1:** Calculation formulae for major vegetation indices.

Vegetation Indices	Formula
Normalized Difference Vegetation Index(NDVI)	NDVI=(NIR−RED)/(NIR+RED)
Renormalized Difference Vegetation Index(RDVI)	$\text{RDVI}=(\text{NIR}-\text{RED}/\sqrt{\text{NIR}/(\text{RED}+1)})$
Normalized Lenticel Index(NLI)	$\text{NLI}=(NIR^{2}-\text{RED})/(NIR^{2}+\text{ GREEN})$
Green Normalized Difference Vegetation Index(GNDVI)	GNDVI=(NIR−GREEN)/(NIR+GREEN)
Ratio Vegetation Index(RVI)	RVI=NIR/RED
Soil Adjusted Vegetation Index(SAVI)	SAVI=1.5(NIR−RED)/(NIR+RED+0.5)
Normalized Difference Greenness Index(NDGI)	NDGI=(GREEN−RED)/(GREEN+RED)
Difference Vegetation Index(DVI)	DVI=NIR−Red
Optimized Soil Adjusted Vegetation Index(OSAVI)	OSAVI=1.16(NIR−RED/(NIR+RED+0.16))
Greenness Index(GI)	GI=GREEN/RED
Modified Simple Ratio(MSR)	MSR=(NIR/RED−1)/((NIR/RED+1))×0.5
Green Red Vegetation Index(GRVI)	GRVI=(GREEN−Red)/(GREEN+Red)
Chlorophyll green Index(CLgreen)	CLgreen=(NIR/GREEN)−1
Weighted Difference Vegetation Index(WDRVI)	WDRVI=(0.2NIR−RED)/(0.2R+RED)
Transformed Vegetation Index(TVI)	TVI=0.5[120(NIR−GREEN)−200(R−GREEN)]
Normalized Difference Water Index(NDWI)	NDWI=(GREEN−NIR)/(GREEN+NIR)

### 2.5 Model construction and evaluation

This study systematically constructed eight machine learning regression models for sorghum yield prediction, including lasso regression, ridge regression, elastic net, random forest, multilayer perceptron, support vector regression, gradient boosting tree, and linear model. The dataset was divided into training and test sets with an 8:2 ratio, and hyperparameter optimization was achieved through 5-fold cross-validation and grid search (**Table 2**). Four spectral bands and 16 yield-related vegetation indices, combined with meteorological data, were selected as input parameters to model and analyze sorghum yield. The model performance evaluation metrics included the coefficient of determination (R^2^) (Equation 1), root-mean-square error (RMSE) (Equation 2), and mean absolute error (MAE) (Equation 3), providing a comprehensive measure of prediction accuracy and stability.

**Table 2:** Parameter settings for different models.

Model	Parameter Settings
RandomForest	n_estimators = 100max_depth = 10max_features = ‘sqrt’min_samples_split = 2min_samples_leaf = 1
LinearRegression	Default parameters
MLPRegressor	hidden_layer_sizes=(64, 32) max_iter=1000 random_state=42
Ridge	alpha=1.0 max_iter=5000
GradientBoosting	n_estimators = 200max_depth = 5learning_rate = 0.1subsample = 0.8min_samples_split = 2
ElasticNet	alpha=0.01 L1_ratio=0.5 max_iter=5000
Lasso	alpha=0.01 max_iter=5000
SVR	kernel = ‘rbf’C = 1.0gamma = ‘scale’epsilon = 0.1degree = 3


(1) $$R^{2}=\frac{\sum_{\text{i}=1}^{\text{n}}({\text{x}}_{\text{i}}-\bar{\text{x}})({\text{y}}_{\text{i}}-\bar{\text{y}})}{\sqrt{\sum_{\text{i}=1}^{\text{n}}{\left({\text{Y}}_{\text{i}}-\bar{\text{Y}}\right)}^{2}}\sqrt{\sum_{\text{i}=1}^{\text{n}}{\left({\text{X}}_{\text{i}}-\bar{\text{X}}\right)}^{2}}}$$

(2) $$\text{RMSE}=\sqrt{\frac{1}{\text{n}}\sum_{\text{i}=1}^{\text{n}}{\left({\text{y}}_{\text{i}}-{\hat{\text{y}}}_{\text{i}}\right)}^{2}}$$

(3) $$\text{MAE}=\frac{1}{\text{n}}\sum_{\text{i}=1}^{\text{n}}|{\text{y}}_{\text{i}}-{\hat{\text{y}}}_{\text{i}}|$$

### 2.6 Analysis of variable importance

To analyze the explanatory power and influence of different input variables in the model, we introduced the SHAP (Shapley Additive Explanations) method to interpret the model output. Key variables were identified by calculating the average marginal contribution of each feature in the prediction process. Additionally, the PDP (Partial Dependence Plot) method was used to plot the partial dependence curves of variables on yield, revealing the trend of the one-factor response of variable changes to model predictions.

### 2.7 Implementation platform and the dataset details

All modeling and analysis were implemented in Python 3.9 using the scikit-learn (v1.2.2), SHAP (v0.41.0), and matplotlib (v3.6.3) libraries. Data preprocessing and geospatial processing were conducted using ArcMap 10.8 and GDAL tools. The deep learning component (MLP) was implemented using TensorFlow (v2.11). All computations were performed on a workstation equipped with an NVIDIA RTX 4080 GPU running Windows 11 64-bit.

## 3 Results and analysis

### 3.1 Validation and evaluation of models

To comprehensively assess the performance of the models in sorghum yield prediction, the test set results were analyzed using three statistical indices: coefficient of determination (R²), root mean square error (RMSE), and mean absolute error(MAE).

**Figure 3:**
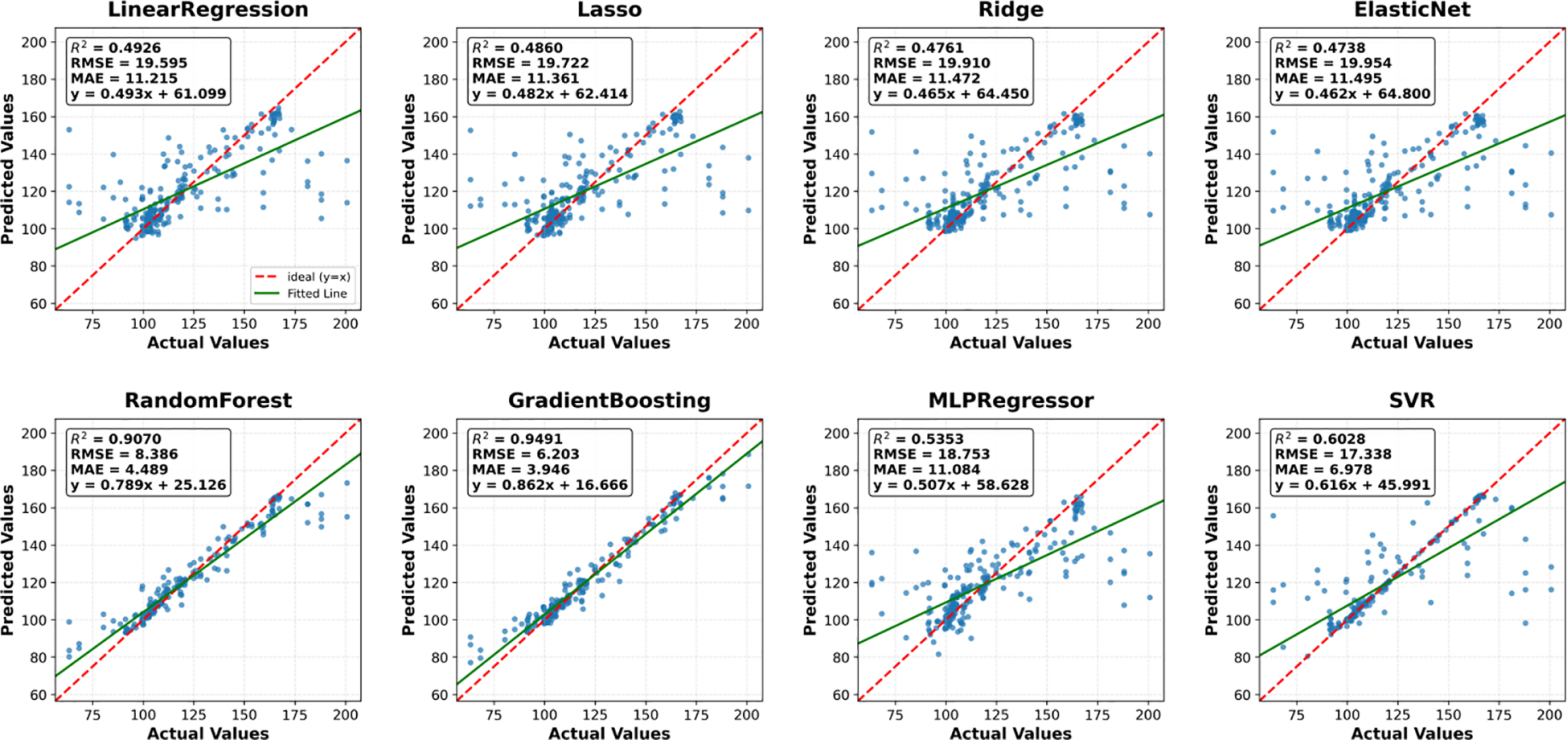
Performance comparison of eight machine learning models for sorghum yield prediction. Each subplot shows measured vs. predicted yield values. The red diagonal line represents perfect prediction. Ensemble methods clearly outperform linear models.

**Figure 4:**
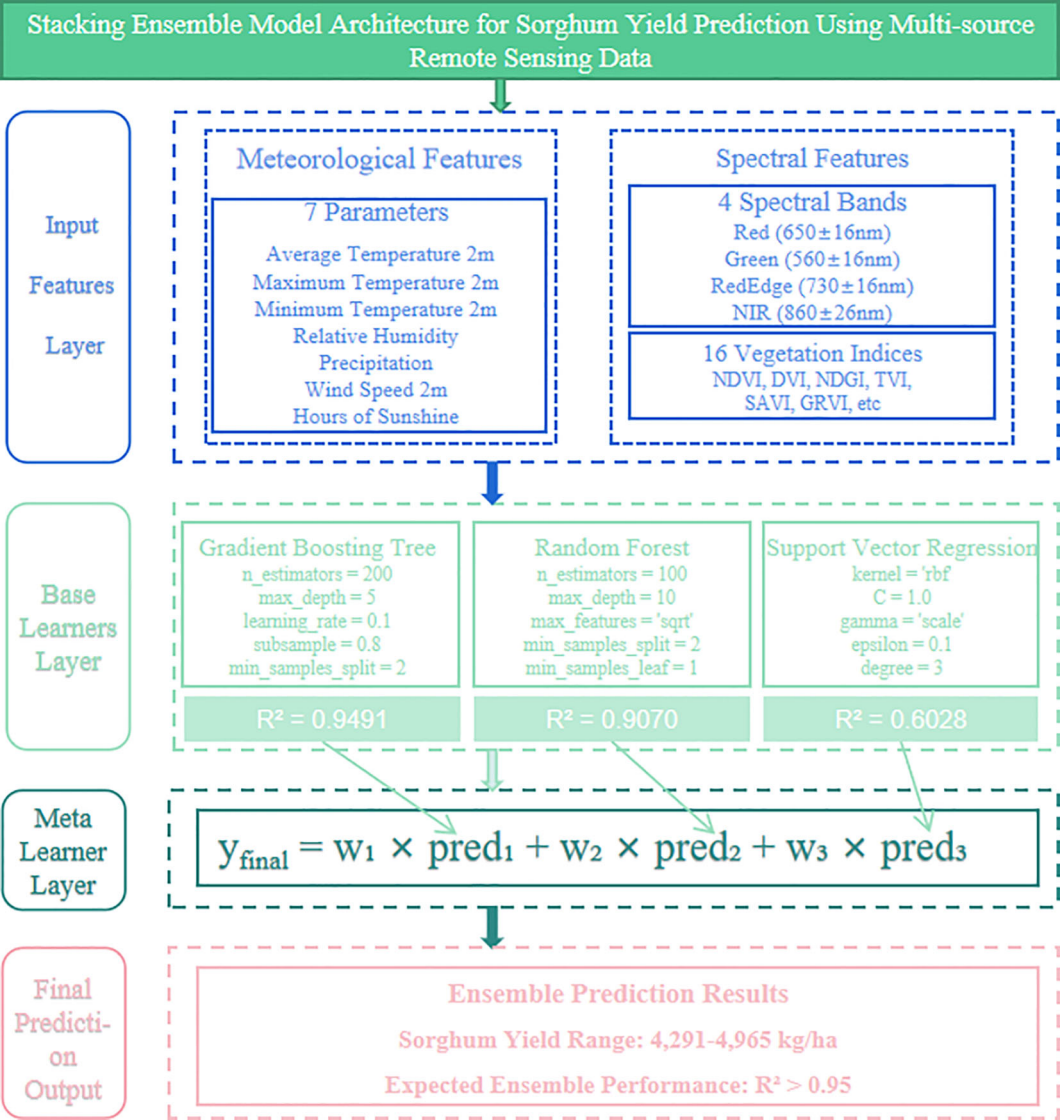
Stacking ensemble model architecture for sorghum yield prediction using multi-source remote sensing data. The four-layer framework integrates spectral and meteorological features through three base learners (Gradient Boosting, Random Forest, SVR) and a linear regression meta-learner. The ensemble approach combines predictions from top-performing models to achieve enhanced yield prediction accuracy (4,291-4,965 kg ha^-1^) with expected R² > 0.95.

Among all eight models, Gradient Boosting performed the best, with an R² of 0.9491, RMSE of 6.2028, and MAE of 3.9460, indicating the strongest fitting ability and prediction accuracy. Random Forest was the next best, with an R² of 0.9070 (**Table 3**). Linear models (e.g., Linear Regression, Lasso, Ridge, ElasticNet) demonstrated weaker overall performance, with an R² below 0.80 and larger errors. The prediction results of MLPRegressor and SVR fluctuate more, with lower accuracy than the ensemble method, and some sample points show noticeable deviations (**Figure 3**). Based on the comprehensive evaluation of R², RMSE, and MAE, the fitting performance was prioritized. The top three models with the best fitting performance were selected to construct a stacked model, which was identified as the optimal solution for sorghum yield prediction (**Figure 4**).

**Table 3:** Comparison of modeling performance across different models.

Model	R²	MAE	RMSE
RandomForest	0.9070	4.4887	8.3860
MLPRegressor	0.5353	11.0836	18.7527
SVR	0.6028	6.9781	17.3378
ElasticNet	0.4738	11.4950	19.9542
Ridge	0.4761	11.4723	19.9104
Lasso	0.4860	11.3613	19.7222
LinearRegression	0.4926	11.2150	19.5946
GradientBoosting	0.9491	3.9460	6.2028

**Figure 5:**
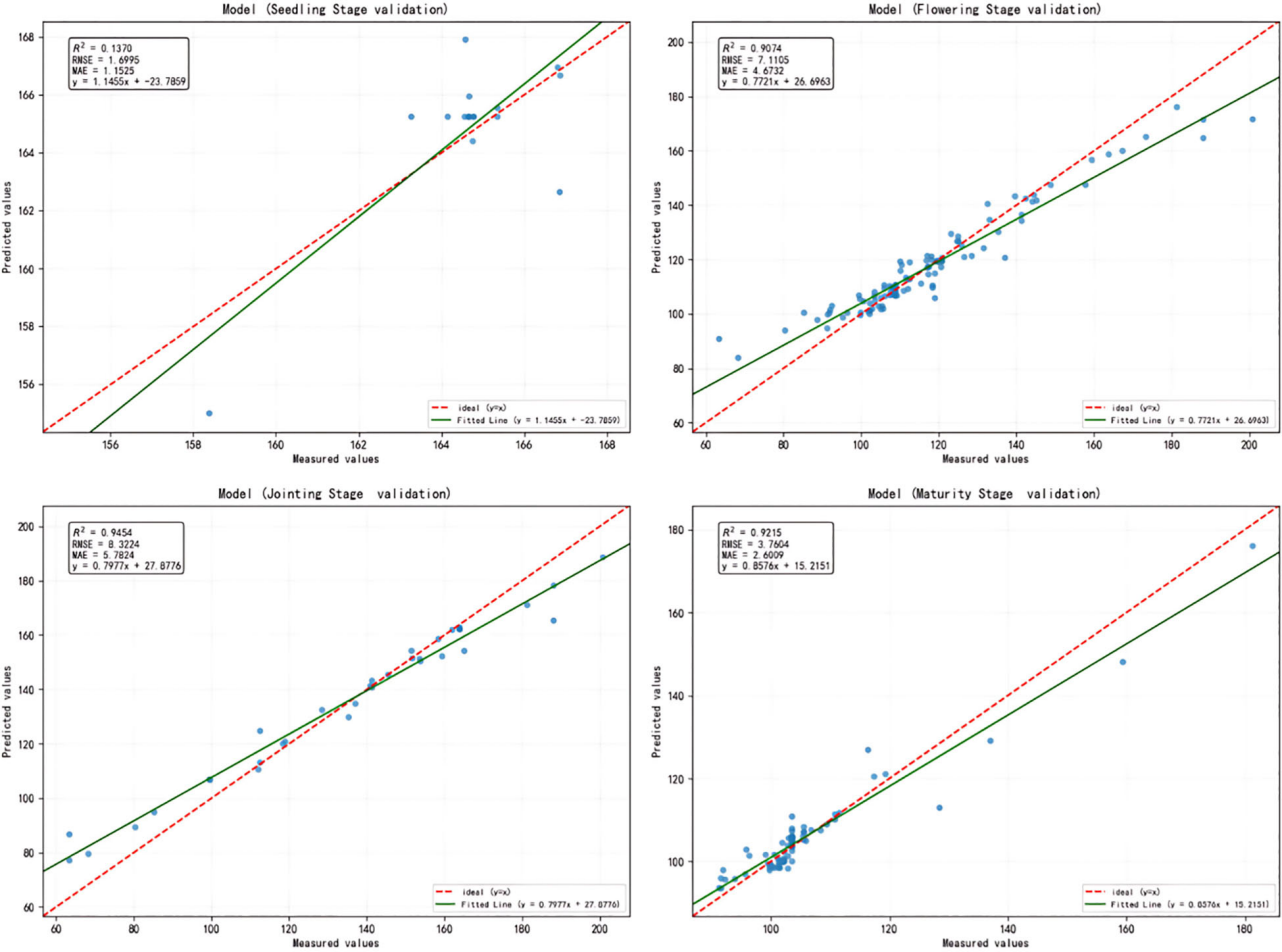
Validation of model predictions for different sorghum growth stages. The red dashed line represents perfect prediction (1:1 relationship), while the green line shows the actual model fit. Jointing stage demonstrates the highest predictive accuracy, followed by maturity and flowering stages, while seedling stage shows poor prediction capability.

To further assess the model’s ability to predict sorghum yield at different fertility stages, the yield prediction results were validated in stages based on seedling emergence, nodulation, flowering, and maturity periods. A comparison of the validation results for sorghum across the four stages of seedling emergence, nodulation, flowering, and maturity revealed significant differences in the contribution of features extracted from each stage to yield prediction. Among these, the features at the jointing stage showed the strongest predictive ability, with generally high model accuracy and a significantly better R² value (R² = 0.9454, **Table 4**) than the other stages. This indicates that remote sensing and environmental information during this period most effectively reflected the final yield differences; The maturity stage performed next best, also showing a stronger predictive effect; The predictive ability at the seedling emergence and maturity stages was relatively weak, likely due to the plants not being fully developed or stabilized at these stages, which meant that yield differences were not yet fully apparent (**Figures 5**).

**Table 4:** Evaluation of model performance across different growth stages.

Model	R²	MAE	RMSE
Seedling Stage	0.1371	1.6995	1.1525
Flowering Stage	0.9075	7.1105	4.6732
Jointing Stage	0.9454	8.3224	5.7824
Maturity Stage	0.9215	3.7604	2.6009

The models were comprehensively evaluated using R², RMSE, and MAE across different growth stages of sorghum, and the jointing stage was identified as the optimal period for sorghum yield prediction.

### 3.2 Analysis of the importance of characterization variables

To explain the model prediction mechanism and identify key features, this study performed a SHAP (Shapley Additive Explanations) value analysis on the stacked model. The SHAP analysis quantitatively assesses the relative importance of each feature and determines how each feature influences model predictions by calculating its contribution magnitude.

**Figure 6:**
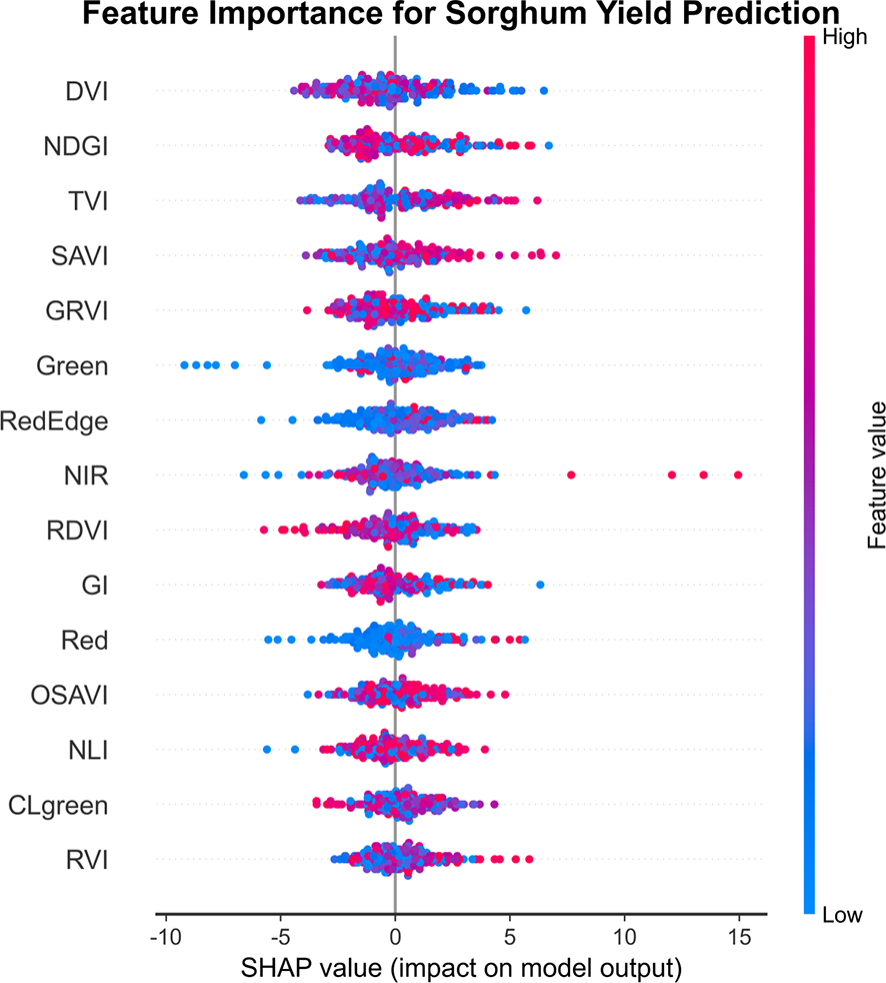
Summary of feature importance based on SHAP values. Features are ranked by mean absolute SHAP value (horizontal axis). DVI and NDGI are the most important predictors, followed by TVI, SAVI, and GRVI. Violin plots show SHAP value distributions, with red dots representing high feature values and blue dots representing low values. Vegetation indices dominate the prediction model compared to individual spectral bands.

**Figure 6** shows that DVI and NDGI are the most influential features in the prediction model, followed by TVI, SAVI, and GRVI, indicating that vegetation indices play a central role in sorghum yield prediction. These spectral indices effectively captured key physiological characteristics of the crop, such as photosynthetic activity, canopy structure, and biomass, which are directly related to yield. The relationship between eigenvalues and SHAP values revealed important patterns. For DVI, higher index values (red dots) were associated with positive SHAP values, indicating that higher DVI values predicted increased yields. In contrast, NDGI exhibited a bi-directional effect, where both very high and very low values potentially led to increased yield predictions. The influence pattern of TVI indicated that moderate values contributed most to yield predictions, while very high values had a weaker effect, reflecting a nonlinear relationship that demonstrates the index’s sensitivity at specific growth stages.

Among the spectral bands, Green, RedEdge, and NIR exhibited significant effects. The red edge band (RedEdge) demonstrated a pronounced nonlinear effect, where high values (red dots) correlated with positive SHAP values, indicating a strong link between red edge reflectance, chlorophyll content, and crop health. Notably, most traits exhibited a wide range of both positive and negative effects on SHAP values, suggesting that the influence of traits on yield is shaped by interactions between their own values and other traits, reflecting the complexity of sorghum yield formation.

**Figure 7:**
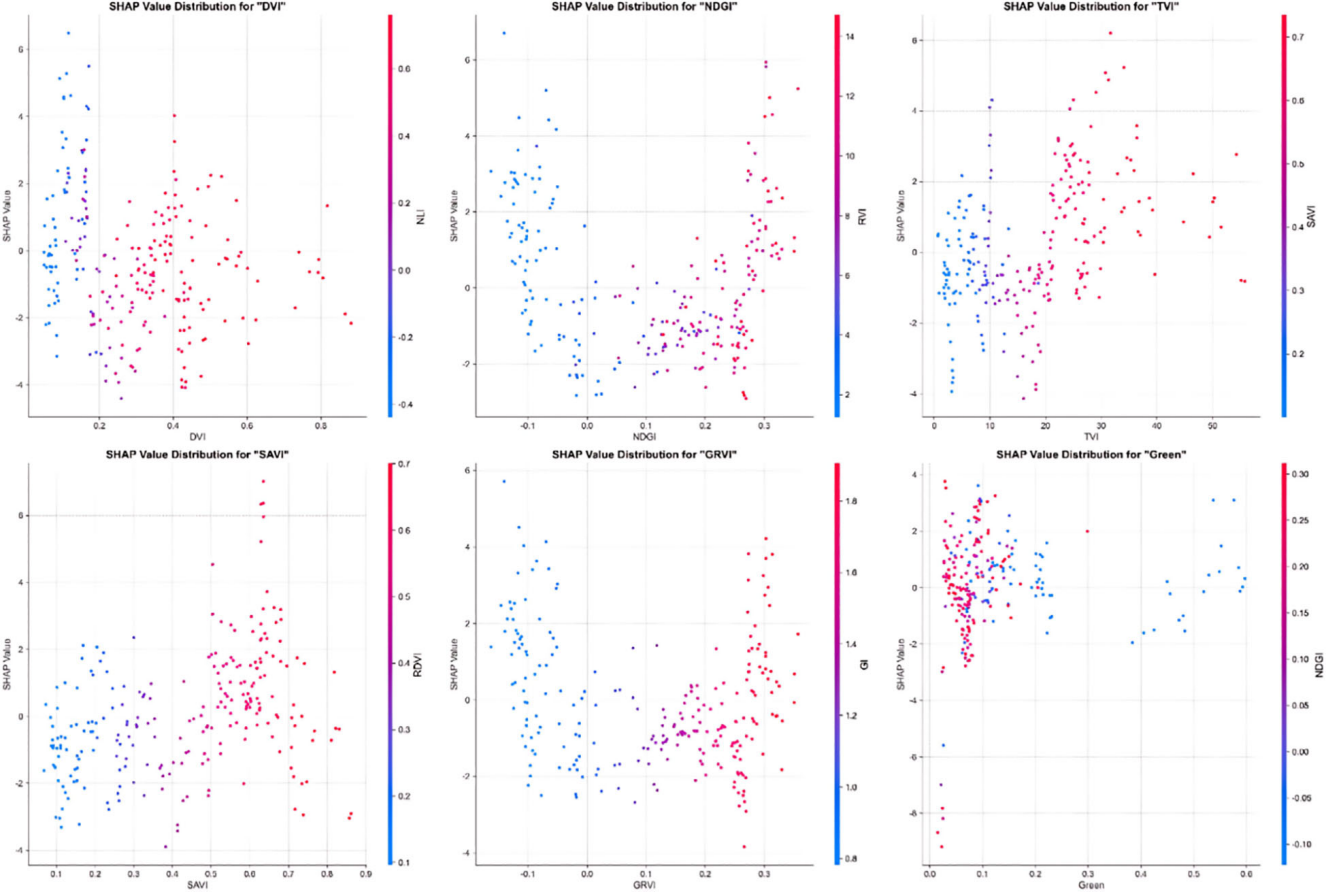
SHAP value plot analyzing the effects of six key features on sorghum yield. Each subplot displays the distribution of SHAP values for different features: DVI, NDGI, TVI, SAVI, GRVI, and Green band. The x-axis represents feature values, y-axis shows SHAP values (impact on model output), and colors indicate feature value magnitude (blue = low values, red = high values). DVI shows the strongest positive impact at high values (>0.6), while TVI demonstrates a clear positive correlation trend with increasing feature values, and NDGI exhibits significant positive effects when values exceed 0.1.

**Figure 7** illustrates the distribution of SHAP values for the six key features in sorghum yield prediction. DVI primarily exhibits a positive contribution in the high-value region (>0.6), while NDGI significantly enhances its positive impact at values greater than 0.1. TVI displays the most distinct positive correlation trend, with its contribution to yield prediction progressively increasing as values rise, especially in the range above 20. SAVI primarily exhibits positive SHAP values in the high-value region (>0.4). GRVI and NDGI display similar but more symmetrical patterns of influence. In contrast, the Green band shows a more complex distribution pattern.

**Figure 8:**
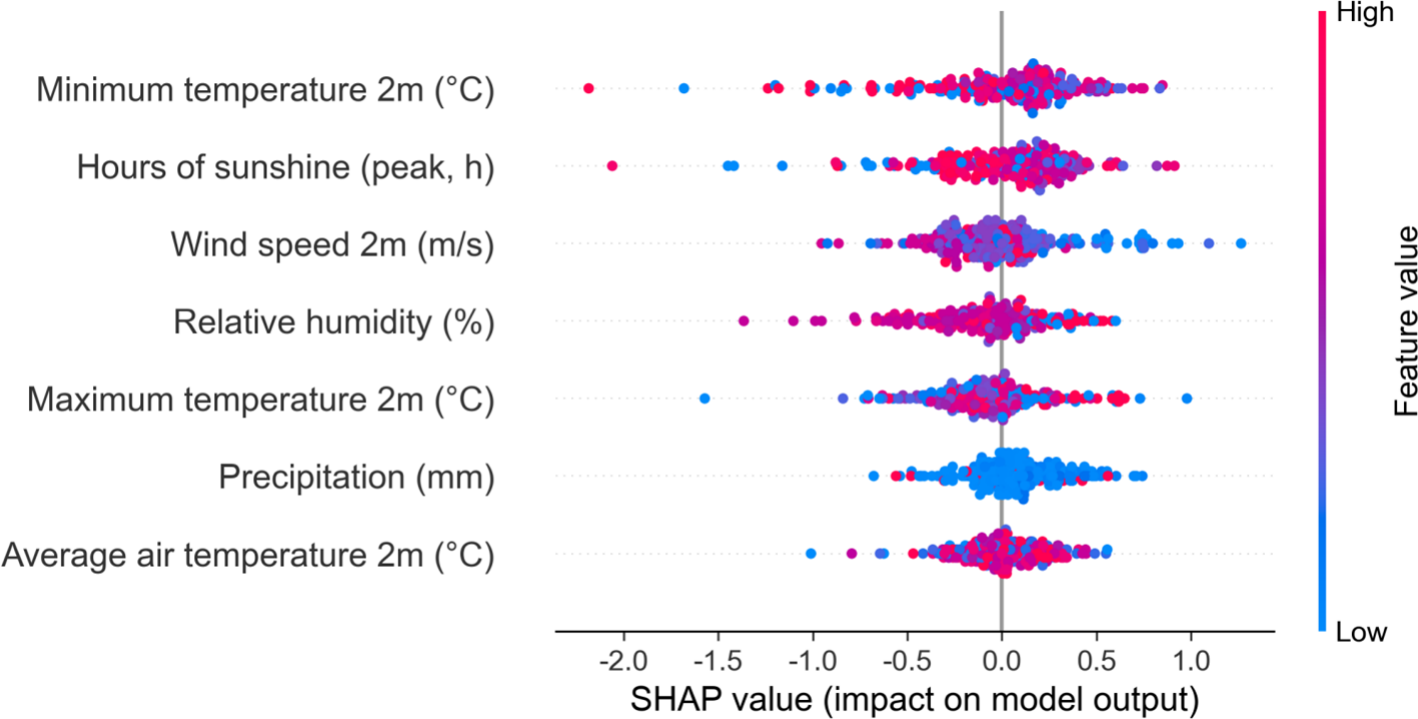
SHAP value plot analyzing the effects of meteorological features on sorghum yield. The plot displays the distribution of SHAP values for seven key meteorological factors: Average air temperature 2m, Precipitation, Maximum temperature 2m, Relative humidity, Wind speed 2m, Hours of sunshine, and Minimum temperature 2m. The x-axis represents SHAP values (impact on model output), y-axis shows different meteorological features, and colors indicate feature value magnitude (blue = low values, red = high values).

**Figure 8** illustrates the distribution of SHAP values for seven meteorological features in sorghum yield prediction. Average air temperature 2m primarily exhibits positive contributions, with higher temperature values (red points) concentrated in the positive SHAP region, indicating that warmer average temperatures enhance yield prediction. Maximum temperature 2m and Minimum temperature 2m display similar positive correlation patterns, where higher temperature values consistently contribute positively to yield prediction, with SHAP values ranging from -0.5 to +1.0. Hours of sunshine shows a relatively symmetrical but predominantly positive influence pattern, with peak sunshine hours (high values) contributing more positively to yield prediction. Wind speed 2m demonstrates a more concentrated distribution around zero SHAP values, suggesting a relatively neutral impact on yield prediction regardless of wind speed levels. In contrast, Relative humidity displays a distinct negative correlation pattern, where higher humidity values (red points) are predominantly associated with negative SHAP values, indicating that excessive humidity reduces predicted yield. Similarly, Precipitation exhibits an inverse relationship, with higher precipitation values (red points) showing negative contributions to yield prediction, while lower precipitation levels (blue points) contribute positively. Among all meteorological features, the temperature-related variables (average, maximum, and minimum temperatures) show the strongest and most consistent positive impacts, while humidity and precipitation demonstrate clear negative relationships with sorghum yield prediction, reflecting the crop’s preference for warm, dry conditions during critical growth periods.

### 3.3 Partial dependence analysis

**Figure 9:**
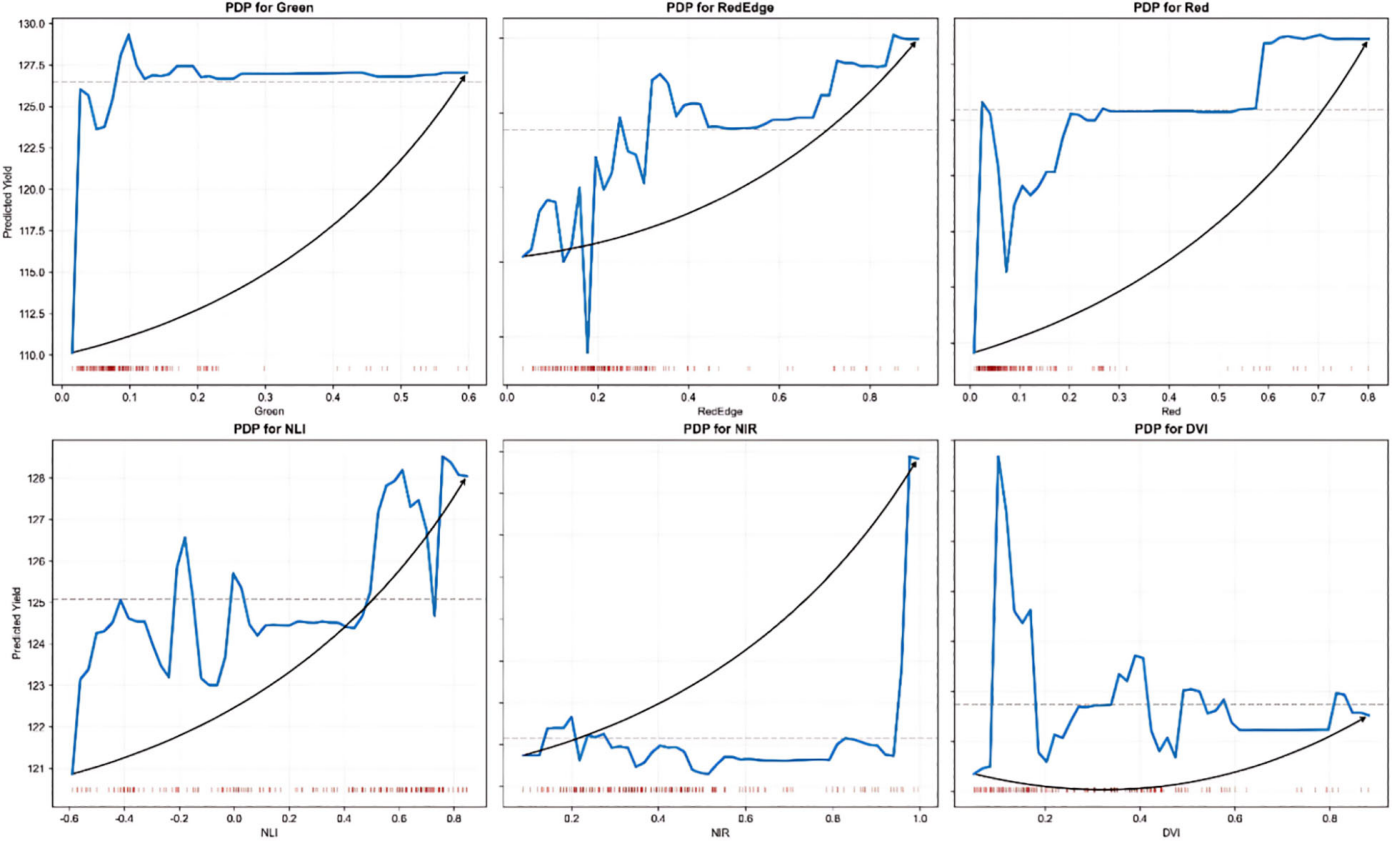
Partial dependency plot analysis of the effects of six key features on sorghum yield. Each subplot shows how individual features influence predicted yield while holding other variables constant: Green and Red bands exhibit positive effects in low-value ranges then stabilize, RedEdge and NIR demonstrate enhanced sensitivity in mid-to-high value ranges, while NLI and DVI display complex nonlinear relationships. The blue curves represent the partial dependence relationship, with red tick marks below indicating data distribution density. These plots reveal the optimal feature value ranges for yield enhancement and provide insights for precision agriculture management decisions.

Partial Dependency Plot (PDP) analysis is a method used to visualize the impact of individual features on the predictive output of a model. It reveals the marginal relationship between the target variable and the predicted outcome while holding other variables constant. A Partial Dependency Plot (PDP) analysis of the six spectral variables (Green, RedEdge, Red, NLI, NIR, and DVI), which have a significant impact on sorghum yield prediction, reveals (**Figures 9**) that the influence of these variables on predicted yields varies significantly across their value ranges, and the overall response trend is nonlinear. Among these variables, the Green and Red bands exhibited a positive effect on yield prediction in the low-value range, which then stabilized. In contrast, NIR and RedEdge were more sensitive to yield enhancement in the middle and high-value ranges, reflecting the stage-specific response of crops to the reflectance of these spectral bands.

### 3.4 Characterization of spatial variability distribution in sorghum yield

#### 3.4.1 Characterization of spatial distribution of sorghum yield

**Figure 10:**
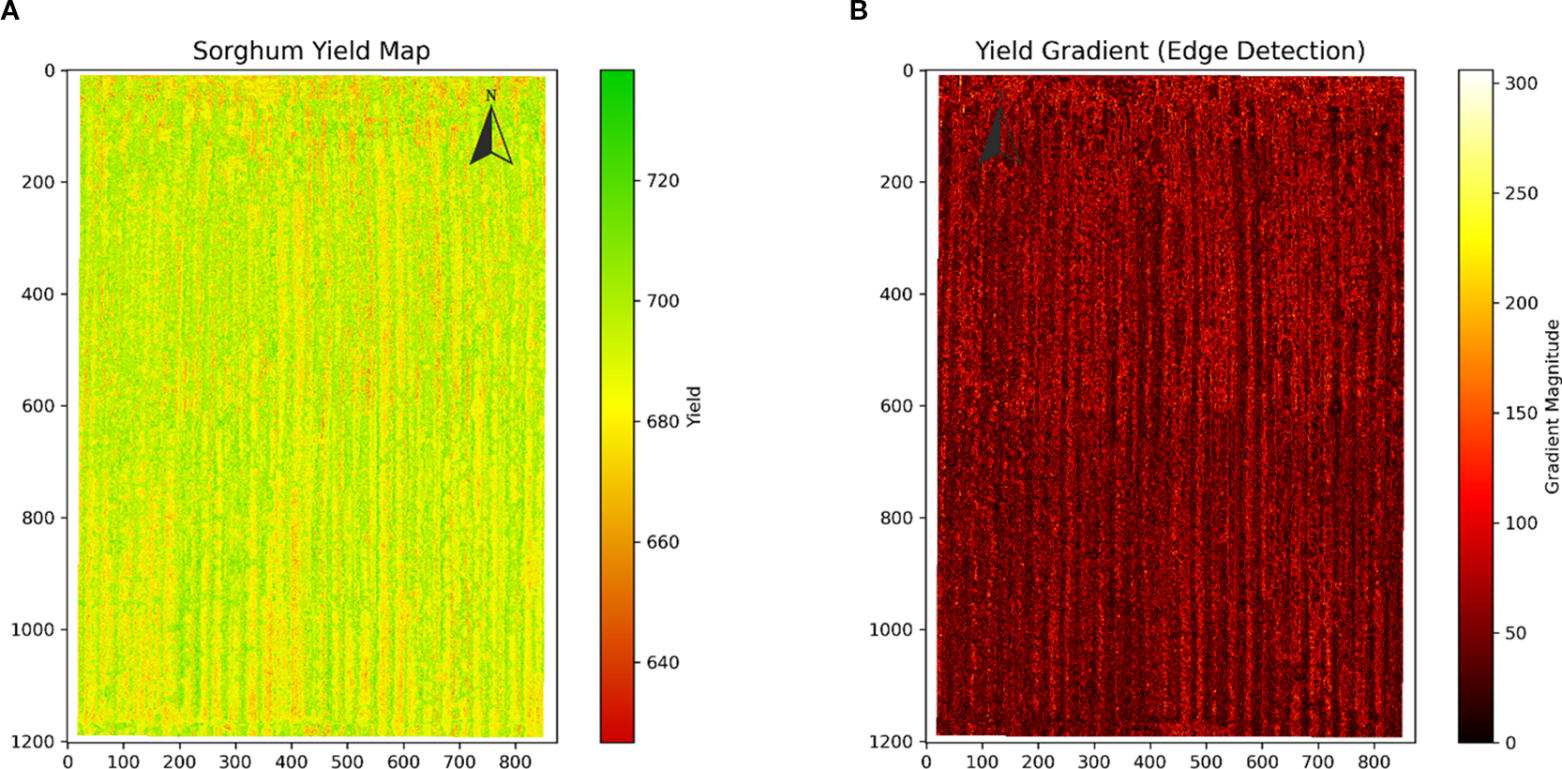
Spatial distribution of yield variability in sorghum. **(A)** Yield distribution map showing distinct north-south oriented striped patterns with high-yield areas (green) and low-yield areas (red-orange) forming regular alternating zones. **(B)** Yield gradient map highlighting regions of rapid yield change (red areas) between different yield zones. The consistent striped pattern suggests systematic field management effects suitable for zone-specific precision agriculture strategies.

Using the optimal yield prediction model established in the previous phase, we mapped the spatial distribution of sorghum yield across the study area. Sorghum yield in the study area exhibited clear spatial heterogeneity with a distinct and regular distribution pattern. Yield values ranged from 630 to 730 pounds per acre, with high-yield areas (green, 700–730 pounds per acre) and medium-yield areas (yellow, 670–690 pounds per acre) forming a north-south oriented pattern of alternating strips (**Figure 10A**). Yield gradient analysis (**Figure 10B**) further confirmed this striped structure, with regions of higher gradient values (red strips) aligning with the boundaries of yield variability, highlighting the characteristic east-west variability in yield.

#### 3.4.2 Spatial autocorrelation characteristics of sorghum yield

**Figure 11:**
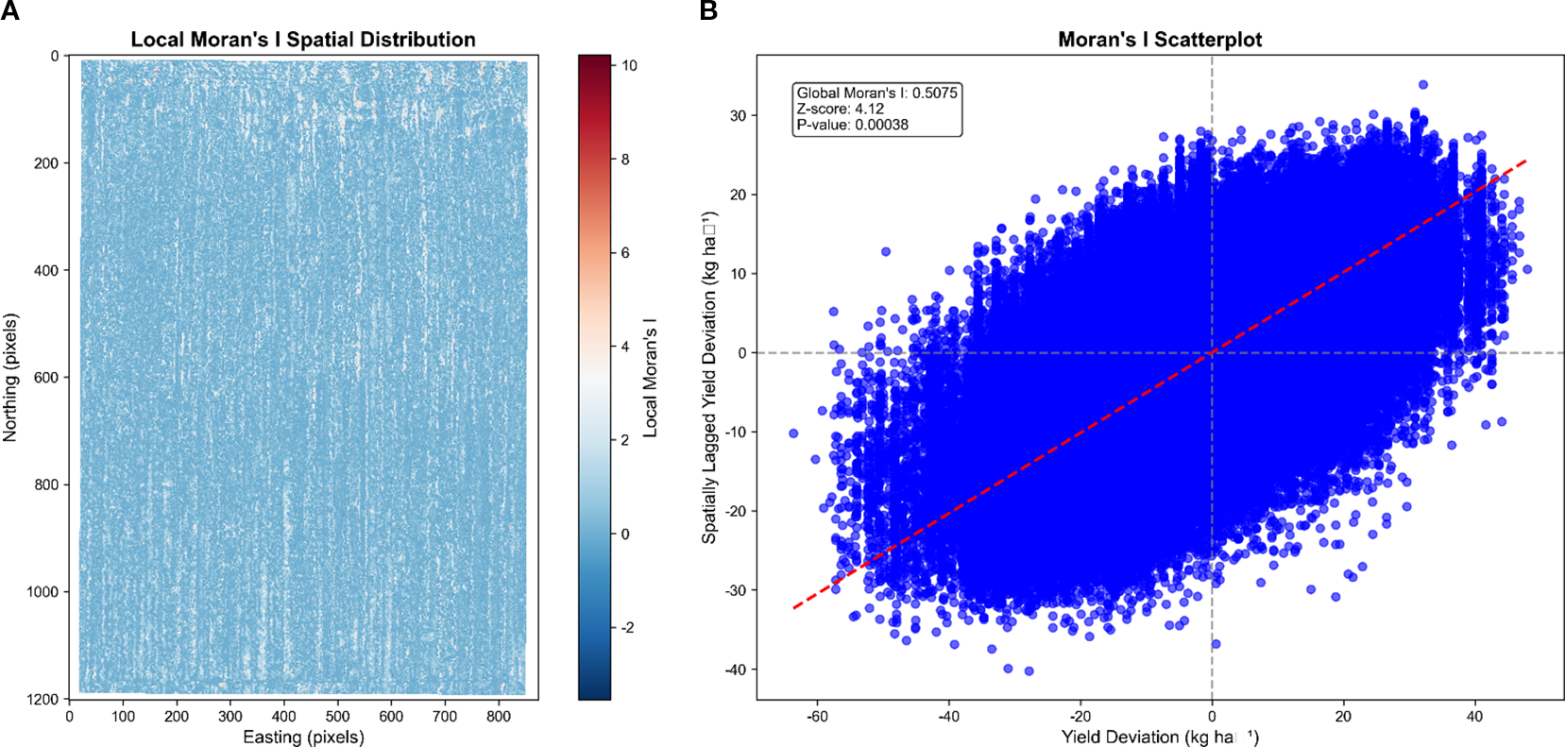
Spatial autocorrelation analysis of sorghum yield. **(A)** Local Moran’s I spatial distribution map showing positive spatial clustering (light blue areas) confirming the striped yield pattern observed in previous maps. **(B)** Moran scatter plot demonstrating positive spatial autocorrelation, with most data points clustering in the first and third quadrants, indicating that high-yield areas neighbor other high-yield areas and low-yield areas cluster together.

Moran’s I (Moran’s Index) is a widely used spatial autocorrelation statistic that measures the degree of similarity among spatial data. It helps determine whether geospatially similar observations are spatially clustered, randomly distributed, or dispersed. The spatial autocorrelation analysis revealed significant spatial dependence of sorghum yield in the study area (**Figure 11**). The global Moran’s I index of 0.5552 indicated moderate positive spatial autocorrelation in yield distribution, meaning that regions with similar yield levels tended to cluster spatially. The spatial distribution map of the local Moran’s I index revealed that the study area was predominantly light blue, displaying a clear striped distribution pattern. This pattern was consistent with the original yield distribution characteristics, further confirming the spatial structure of yield distribution.

The Moran scatter plot further confirmed the spatial clustering characteristics of yield, with most scatter points concentrated in the first quadrant (high-high aggregation) and the third quadrant (low-low aggregation). This distribution indicates that areas surrounding high-yield regions tend to be high-yield areas, while areas around low-yield regions tend to be low-yield areas. The higher density of scatter points along the red trend line further validated the significant positive correlation between yield values and their spatial lag values.

#### 3.4.3 Spatial frequency characteristics of sorghum yield

**Figure 12:**
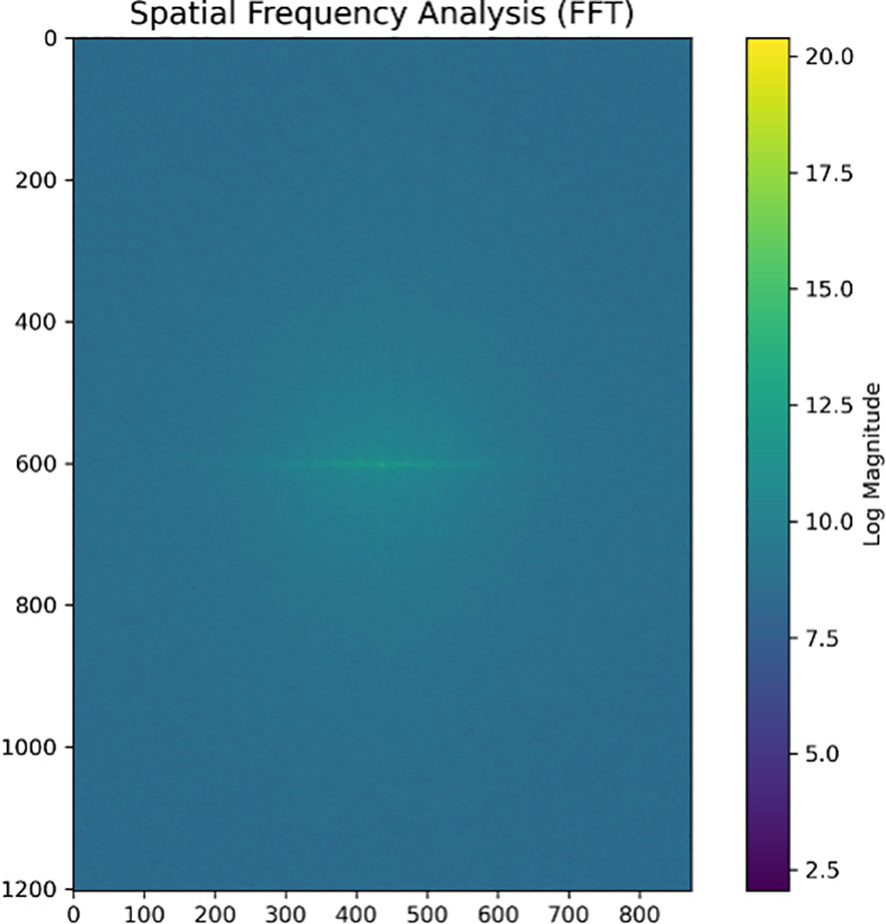
Spatial frequency analysis map. The frequency spectrum reveals the periodic characteristics of yield spatial patterns, with the color scale representing frequency magnitude intensities. The analysis confirms the regular spatial periodicity observed in yield distribution maps, providing frequency domain evidence for the systematic striped patterns identified in the field layout.

Spatial frequency analysis using Fast Fourier Transform (FFT) (**Figures 12**) revealed the periodic structural characteristics of sorghum yield data. The presence of horizontal bright bands in the central region of the spectrogram indicates regular periodic variations in the vertical direction of the study area. This frequency feature aligns with the previously observed striped pattern of yield distribution, further confirming the direction-dependent nature of yield distribution from a frequency domain perspective. The relatively concentrated distribution of frequency intensities suggests a more regular spatial periodicity of yield. This pattern may reflect the systematic effects of field management activities, such as agricultural practices aligned along specific directions, including mechanical operations, irrigation layout, or planting methods.

## 4 Discussion

This study achieved a series of significant findings in sorghum yield prediction by integrating UAV multispectral technology with meteorological data. Integrated learning models, such as Gradient Boosting and Random Forest, demonstrated the highest performance in sorghum yield prediction, achieving R² values of 0.93 and 0.91, respectively, significantly outperforming traditional linear models. These findings align with the results of studies by Zhou et al. (Zhou et al., 2025) and Kumar et al. (Kumar et al., 2023), which similarly reported superior performance of image-based deep learning models and Random Forest in crop yield prediction. These studies found that image-based deep learning models and Random Forest algorithms significantly outperformed feature-based machine learning models in predicting crop yields, effectively capturing the complex nonlinear relationship between crop growth and yield. Feature importance analysis highlighted the central role of DVI and NDGI in yield prediction, followed by TVI, SAVI, and GRVI, indicating that vegetation indices are highly valuable for sorghum yield prediction. Tanabe et al. (Tanabe et al., 2023) applied convolutional neural networks and UAV multispectral imagery for yield prediction of winter wheat, and similarly found that specific vegetation indices were significantly correlated with yield. Van Klompenburg et al. (Van Klompenburg et al., 2020), through a systematic literature review, highlighted that spectral vegetation indices play a key role in crop yield prediction, effectively reflecting crop health and biomass accumulation. Compared to traditional ground sampling methods, UAV multispectral technology offers high-resolution, non-invasive crop growth monitoring, significantly reducing monitoring costs and improving efficiency, providing essential technical support for the development of precision agriculture.

Recent comparative studies have demonstrated the broad effectiveness of machine learning approaches across diverse agricultural applications, validating our methodological approach. Guo et al. (Guo et al., 2023)found that ensemble methods, particularly Random Forest and Gradient Boosting, consistently outperform individual algorithms for maize yield prediction using UAV hyperspectral images, directly supporting our Gradient Boosting results (R² = 0.9491). Similarly, Guo et al. (Guo et al., 2022; Guo et al., 2024) established the importance of multi-spectral vegetation indices for crop physiological parameter estimation and yield prediction in maize, paralleling our identification of DVI and NDGI as key predictors, while Guo et al. (Guo et al., 2021) demonstrated that integrating phenological and climatic data significantly improves rice yield prediction accuracy, consistent with our systematic growth stage analysis revealing jointing (R² = 0.9454) and flowering (R² = 0.9075) as optimal monitoring periods. However, our study extends these findings to drought-tolerant sorghum under arid conditions with the novel integration of spatial autocorrelation analysis (Moran’s I = 0.5552), addressing the specific challenges of water-limited agriculture where traditional approaches may be insufficient.

Analysis of the predictive ability at different growth stages showed that features at the nodulation and flowering stages contributed the most to yield prediction, with R² values of 0.9454 and 0.9075, respectively. In contrast, the emergence stage exhibited relatively weak predictive ability (R² = 0.1371). This finding is of practical significance, indicating that the emergence stage is a critical phase of sorghum growth during which the plant rapidly develops its basic architecture. The growth status in this period directly influences yield formation in later stages. This is consistent with the findings of Samera et al. (Shammi et al., 2024) and Camenzind et al. (Camenzind and Yu, 2024), who reported that multispectral data during critical growth stages showed a stronger correlation with final yield. Li et al. (Li et al., 2019) systematically compared the yield predictive ability of maize across all growth stages and similarly confirmed that canopy characteristics during the mid-growth stage were most indicative of final yield formation. Camenzind et al. (Camenzind and Yu, 2024) also emphasized that the mid-growth stage of a crop can reflect environmental and management impacts from earlier stages and predict the potential for yield formation in later stages, making it the optimal time window for yield prediction. This finding is crucial for optimizing monitoring timing and enhancing prediction efficiency, allowing limited monitoring resources to be concentrated on key growth stages, such as nodulation and flowering, thereby obtaining the most valuable data for prediction.

Spatial autocorrelation and frequency analyses revealed the spatial distribution characteristics of sorghum yield within the study area. The Moran’s I index (I = 0.5552) indicated moderate positive spatial autocorrelation in yield distribution, forming a clear striped pattern. This analytical method effectively identifies high-yield and low-yield areas in the field, providing a scientific basis for precision management (Reynolds, 1970). The spatial distribution of the local Moran’s I index further confirmed the spatial structural characteristics of yield distribution, primarily manifesting in high-high and low-low aggregation patterns. Fast Fourier Transform (FFT) spectral analysis revealed regular periodic variations in the vertical direction of the study area. This method effectively identifies the main frequency patterns of spatial distribution by converting spatial-domain data into frequency-domain data. Maestrini and Basso (2018) emphasized that long-term analysis of spatial and temporal patterns of crop yields can reveal the characteristics of yield variability, identify stable high-yield and low-yield areas, and highlight regions with significant interannual fluctuations. They found that historical yield maps were the best predictors of spatial variability for stable spatial patterns over time, while intra-seasonal remotely sensed imagery was most effective for predicting spatial patterns in areas strongly influenced by weather with significant interannual fluctuations. These findings are highly consistent with our results, confirming the significant value of spatial autocorrelation analysis and frequency analysis in understanding the spatial variability of farmland yield.

Despite the promising results of this study, several limitations remain. First, this study relies on data from a single growing season without cross-annual validation, which may not fully capture the impact of climate fluctuations on the prediction model. Second, the current model primarily relies on spectral information and meteorological data, without fully accounting for soil characteristics, management practices, and other influencing factors. Third, the study area is relatively limited, and the generalization ability of the models requires further validation. Li et al. (Li et al., 2025) highlighted that applying yield prediction models across different climatic regions presents significant challenges, emphasizing the need for developing transfer learning methods with greater generalization capability. Lobell et al. (Lobell, 2013) emphasized the significant potential of satellite data for crop yield gap analysis, while noting that many potential application areas remain unexplored.

In addition, this study relies on data from a single growing season (2024) and a single experimental site, which may limit the model’s generalization across years and regions. To address this limitation, future work will involve multi-year validations and cross-regional testing in diverse agro-ecological zones. These efforts aim to enhance the robustness, transferability, and practical applicability of the proposed framework in broader agricultural scenarios.

Future research should prioritize the application of multi-source data fusion, deep learning, and transfer learning methods. Mariano et al. (Mariano and Mónica, 2021) proposed a data-intensive spatial interpolation algorithm based on Random Forests (QRFI), which can be effectively applied to spatial interpolation of large-scale yield data and the assessment of prediction uncertainty. Camps-Valls et al. (Camps-Valls et al., 2021) demonstrated that combining vegetation indices from different growth stages can enhance absolute yield prediction. However, a single-index model outperformed in spatial pattern prediction. Future studies should consider integrating multi-source data, including thermal infrared, radar, and other remote sensing data, as well as ground sensor network data, to establish a more comprehensive monitoring system. Incorporating deep learning and computer vision technologies to enhance feature extraction capabilities and improve prediction accuracy; Conducting multi-year and multi-regional validation studies to strengthen the model’s robustness and applicability; Integrating the yield prediction model with a decision support system to establish a closed-loop framework from monitoring to management, providing an end-to-end solution for precision agriculture. Furthermore, with the continuous improvement of satellite remote sensing resolution and increased observation frequency, the integration of UAV and satellite remote sensing data can be further explored. This approach enables large-scale, high-frequency, and cost-effective crop growth monitoring and yield prediction, providing a scientific basis for regional agricultural management and ensuring food security.

## 5 Conclusion

Accurate sorghum yield prediction is critically important for ensuring food security in arid and semi-arid regions, where climate change and water scarcity pose increasing challenges to agricultural sustainability. Traditional yield estimation methods are inadequate for the precision agriculture requirements of drought-tolerant crops, necessitating the development of advanced remote sensing and machine learning approaches.

This study predicted and forecasted sorghum yield using UAV multispectral data combined with fused meteorological data, leading to the following main conclusions:

1. We successfully developed a UAV multispectral-based yield prediction system that achieved exceptional accuracy (R² = 0.9491 for Gradient Boosting), demonstrating the superiority of ensemble learning methods over traditional linear approaches for complex agricultural data.
2. Our systematic analysis identified critical vegetation indices (DVI, NDGI, TVI) as key predictors, providing actionable insights for variable-rate management and precision irrigation strategies.
3. The identification of jointing and flowering stages as optimal monitoring periods offers practical guidance for reducing data collection costs while maintaining high prediction accuracy, enabling more efficient resource allocation in precision agriculture.
4. The spatial autocorrelation analysis (Moran’s I = 0.5552) revealed systematic yield patterns that inform site-specific management decisions and precision agricultural practices.

These findings have significant practical implications for sustainable agriculture in water-limited environments. The developed framework enables farmers and agricultural managers to optimize irrigation timing, implement variable-rate fertilization, and make informed management decisions that enhance both productivity and resource use efficiency.

## Data Availability

The original contributions presented in the study are included in the article/Supplementary Material. Further inquiries can be directed to the corresponding author.
